# Compatibility of *Azospirillum brasilense* with Pesticides Used for Treatment of Maize Seeds

**DOI:** 10.1155/2020/8833879

**Published:** 2020-07-10

**Authors:** Mariana S. Santos, Artur B. L. Rondina, Marco A. Nogueira, Mariangela Hungria

**Affiliations:** ^1^Embrapa Soja, C.P. 231, Londrina, 86001-970 Paraná, Brazil; ^2^Department of Biochemistry and Biotechnology, State University of Londrina, C.P. 10.011, Londrina, 86057-970 Paraná, Brazil

## Abstract

Seed treatment with chemical pesticides is commonly used as an initial plant protection procedure against pests and diseases. However, the use of such chemicals may impair the survival and performance of beneficial microorganisms introduced via inoculants, such as the plant growth-promoting bacterium *Azospirillum brasilense*. We assessed the compatibility between the most common pesticide used in Brazil for the treatment of maize seeds, composed of two fungicides, and one insecticide, with the commercial strains Ab-V5 and Ab-V6 of *A. brasilense*, and evaluated the impacts on initial plant development. The toxicity of the pesticide to *A. brasilense* was confirmed, with an increase in cell mortality after only 24 hours of exposure *in vitro*. Seed germination and seedling growth were not affected neither by the *A. brasilense* nor by the pesticide. However, under greenhouse conditions, the pesticide affected root volume and dry weight and root-hair incidence, but the toxicity was alleviated by the inoculation with *A. brasilense* for the root volume and root-hair incidence parameters. In maize seeds inoculated with *A. brasilense*, the pesticide negatively affected the number of branches, root-hair incidence, and root-hair length. Therefore, new inoculant formulations with cell protectors and the development of compatible pesticides should be searched to guarantee the benefits of inoculation with plant growth-promoting bacteria.

## 1. Introduction

Global population reached 7.6 billion people in 2017 and, according to the predictions, will increase to 11.2 billion by 2100 [1]. In this context, it is necessary to produce more food but also to search for production strategies resulting in minimum environmental impact. The inoculation of crops with plant growth-promoting bacteria (PGPB), especially those that can contribute to the reduction of chemical fertilizers usage, attends to these concepts of agricultural sustainability [2].

Several benefits have been attributed to the inoculation with *A. brasilense*, including the supply of N by the biological nitrogen fixation (BNF) process [3, 4], stimulation of root growth [5, 6], phosphate solubilization [7], and increased tolerance to abiotic [8, 9] and biotic [10] stresses. In the case of the Brazilian commercial strains of *A. brasilense* Ab-V5 and Ab-V6, the main effects have been attributed to the production of phytohormones [3, 4].

The use of fungicides and insecticides for seed treatment has been broadly employed in Brazil, with maize representing the second crop that receives more pesticides, after soybean (*Glycine max* (L.) Merr.) [11]. However, the use of inoculants with *A. brasilense* for the maize crop is also impressively increasing, for example, in Brazil, from zero to 7 million doses per year^−1^ in less than a decade, and agrochemicals could affect bacterial survival and plant growth. For example, in the *Bradyrhizobium-*soybean symbiosis, the incompatibility with pesticides may cause lower nodulation and, consequently, lower BNF rates [12, 13].

There is still little information about the effects of pesticides used for the treatment of maize seeds on *A. brasilense* survival and plant growth. As seed treatment is widespread in the maize cropping, studies on the impact of pesticides in *A. brasilense* are necessary to understand the effects on the bacterium and on the mechanisms of plant growth promotion.

## 2. Materials and Methods

### 2.1. Microorganisms, Fungicides, and Insecticides

The experiments were performed with *Azospirillum brasilense* strains Ab-V5 (=CNPSo 2083) and Ab-V6 (=CNPSo 2084). The strains are deposited at the “Diazotrophic and Plant Growth-Promoting Bacteria Culture Collection of Embrapa Soja” (WFCC Collection #1213, WDCM Collection #1054) in Londrina, State of Paraná, Brazil. These two strains have been broadly used in the production of commercial inoculants for the maize crop in Brazil since 2009 [3]. Depending on the analysis, the strains were cultured together or separately.

The pesticide used was Standak™ Top (BASF), broadly employed for the treatment of maize seeds. It consists of a mixture of one insecticide (Fipronil 25%) and two fungicides (pyraclostrobin 2.5% and thiophanate-methyl 22.5%). The recommended dose is of 2 mL per kg of maize seeds.

### 2.2. Recovery of *A. brasilense* from Maize Seeds Treated with Pesticides

The recovery of *A. brasilense* strains Ab-V5 and Ab-V6 from maize seeds treated with the pesticide was based on the method described by Santos et al. [14]. The inoculum was prepared in the modified liquid DYGS medium (composed of glucose, 2 g; malic acid, 2 g; Bacto peptone, 1.5 g; yeast extract, 2 g; K_2_HPO_4_, 0.5 g; MgSO_4_·7H_2_O, 0.5 g; and glutamic acid, 1.5 g; pH 6.8), incubated at 28°C, and shook at 120 rpm until the cell concentration of 10^8^ CFU·mL^−1^. Seeds were previously hydrated in distilled sterile water for 2 h and treated with Standak™ Top at the dose of 2 mL·kg^−1^, or left untreated; both treatments were left to dry at room temperature for 2 h. The inocula were diluted in distilled sterile water prior to seed inoculation (1 : 2, v/v). Treated and nontreated seeds were then inoculated with *A. brasilense* strains Ab-V5 and Ab-V6, applied to the seeds at the rate of 5 mL·kg^−1^, corresponding to 10^5^ cells seed^−1^. Cell recovery from seeds was performed 30 minutes and 24 h after inoculation.

Three biological samples of 100 seeds each were transferred to sterile Erlenmeyer flasks containing 100 mL of sterile water with Tween 80 (0.4 mL·L^−1^). The flasks were submitted to horizontal agitation at 150 rpm for 30 min, resulting in the dilution 10°. One-milliliter aliquot of the recovery sample was then transferred to a sterile flask with 9 mL of 0.85% physiological solution, resulting in the 10^−1^ dilution. The flask was shaken on a vortex. The process was repeated until the 10^−4^ dilution was obtained. 100 *μ*L aliquot of each replicate of each dilution was spread into Rojo Congo (RC) solid culture medium [15] [composed of, per L, DL-malic acid, 5 g; yeast extract, 0.5 g; K_2_HPO_4_, 0.5 g; MgSO_4_·7H_2_O, 0.2 g; NaCl, 0.1 g; KOH, 4.8 g; FeCl_3_.6H_2_O, 0.015 g; Congo Red, 15 mL (solution at 0.25 mg 100 mL); and agar, 20 g; pH 7.0], with vancomycin (0.1 g·L^−1^) to avoid seeds' contaminants, followed by incubation for 5 days at 28 ± 2°C. The recovery of viable cells was based on the counting of colony-forming units (CFU). Each treatment was performed in triplicate.

The experiment was performed on a completely randomized design (CRD), and the data were submitted to the analysis of variance (ANOVA), followed by the comparison of means by Tukey's test at *p* < 0.05 with the statistical software.

### 2.3. Seed Germination and Seedling Vigor of Maize Treated with Pesticides and Inoculated with *A. brasilense*

In order to evaluate the compatibility between the pesticide and the *A. brasilense* inoculant, as well as the effects on seed germination and initial growth of maize seedlings, maize seeds (Agroceres AG9010 PRO VT) were surface-disinfected and, depending on the treatment, were treated or not with Standak™ Top and with a mixture of strains Ab-V5 and Ab-V6 of *A. brasilense.* Four treatments were evaluated: NPNI (control with no pesticide, no inoculant); WPNI (with pesticide, no inoculant); NPWI (no pesticide, with inoculant); and WPWI (with pesticide, with inoculant). Each treatment consisted of three replicates, each with 12 seeds and the experiment was performed in a completely randomized design (CRD).

Seed surface disinfection was performed in ethanol 70% for 1 min, hypochlorite 10% for 5 min and then washed in sterile distilled water five times. First, the seeds of the treatments with the pesticide were treated with Standak™ Top at a rate of 2 mL·kg^−1^ of seeds, as recommended by the manufacturer. Seeds were allowed to dry at room temperature for 2 h.

For each treatment, a tray was disinfected with 10% hypochlorite and then left under laminar flow UV light for 20 min. Following, three Germitest papers (JProlab^®^) previously autoclaved were placed in the tray and moistened with sterile water. The seeds were scattered over two papers, forming 12 columns. Following, the seeds of the treatments with inoculation each received the inoculant of *A. brasilense* at a final concentration of 10^5^ cells of *A. brasilense* per seed and the third paper was placed over the seeds. The papers were rolled up; their ends were tied with elastic bands and incubated in a seed germinator DeLeo^®^ at 25°C and 45 ± 5% of relative air humidity for seven days.

After this period, seed germination rate and seedling vigor parameters, including total length of the seedlings (from the tip of the primary root to the top of the primary leaf), the length of the shoot, and the length of the main root axis, were evaluated with the aid of a ruler with a graduation in mm [16]. The average length was obtained by summing the measurements taken from each normal seedling at each repetition and then dividing by the number of normal seedlings measured.

The experiment was performed in a completely randomized design (CRD) with the treatments arranged in a factorial scheme (2 × 2), involving presence/absence of pesticide and presence/absence of inoculation. Statistical analysis was performed using the RBIO statistical software. Data obtained were evaluated for normality and variance homogeneity, followed by the analysis of variance (ANOVA) by Tukey's test at 5% probability.

### 2.4. Greenhouse Experiment

A greenhouse experiment was carried out to assess the effects of the pesticide used together with the inoculant on the initial development and root morphology of maize. Plants were grown in modified Leonard jars [17] containing 750 cm^3^ of sterilized substrate, consisting of a mixture of sand and pulverized coal (3 : 1, v/v), containing sterile nutrient solution with all macronutrients and micronutrients [18]. The experiment consisted of the same four different treatments described before: NPNI, WPNI, NPWI, and WPWI. The experimental design was performed in a completely randomized design (CRD) with treatments arranged in a factorial scheme (2 × 2), involving presence/absence of pesticide and presence/absence of inoculation with *A. brasilense*, with five replicates.

Maize seeds (Agroceres AG-9010) were surface-sterilized with 70% ethanol and 3% sodium hypochlorite [17]. For the treatments WPNI and WPWI, the seeds were treated with Standak™ Top (2 mL per kg of maize seeds) and left to dry for two hours. For the treatments NPNI and NPWI, this step was not performed. The seeds of the treatments NPWI and WPWI were inoculated with a mixture of Ab-V5 and Ab-V6, ensuring a concentration of 10^5^ cells seed^−1^. Three seeds were sown and thinned to one plant per jar four days after emergence. Nutrient solution was applied as needed, and the temperature and humidity at the greenhouse were controlled by means of air conditioners (25° ± 5°C and 80 ± 5%, respectively).

Thirty-eight days after emergence, plant height (cm) and culm diameter (mm) were assessed with the aid of a ruler and digital caliper. Plants were harvested and the shoots were separated from the roots. The shoots were oven-dried at 60°C until constant dry weight. The roots were washed with running water until completely clean. Approximately, 1 g of fresh roots from each sample was separated and stained in methylene blue (1%) solution for 1 min and washed again in water and scanned with Epson Perfection V370 Photo^®^ for further morphological analysis. The remaining roots were oven-dried under the same conditions as the shoot.

The scanned root images were analyzed using GiA Roots^®^ software to assess specific length (m·g^−1^), weighted average diameter (mm), tissue density, and volume (cm^3^). The value determined in each scanned root fragment was estimated for the total fresh mass of the root system.

Approximately, 0.1 g of fresh fine roots was obtained from each sample, stored in FAA solution (90% ethanol 70%, 5% formaldehyde, and 5% acetic acid) and used for assessment of root-hair incidence, root-hair length, and number of root branches. The number of root branches was counted using a stereomicroscope at 30X magnification to estimate the number of lateral roots. Root-hair incidence was determined by the presence or absence of root hairs on 150 fine-root intersections using the gridline method [19]. Root-hair length was assessed measuring 50 root hairs in fine-root segments using a microscope at 100X magnification with an ocular micrometer.

The dataset was first evaluated for normality and variance homogeneity. Means were compared using the analysis of variance (ANOVA) followed by Tukey's test at 5% probability. All analyses were performed in the software RBIO®.

## 3. Results and Discussion

**Figure 1:**
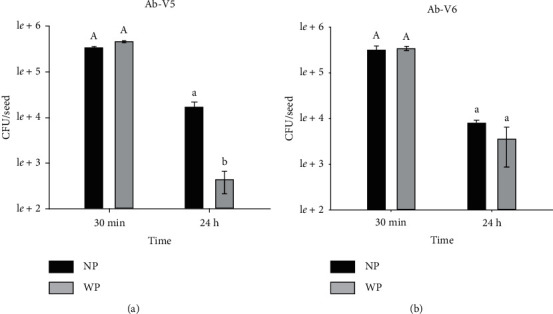
Recovery of *Azospirillum brasilense* cells (expressed in CFU seed^−1^) from maize seeds treated (WP) or not (NP) with pesticide 30 minutes and 24 h after inoculation. Data represent the means of three biological replicates, each with three technical replicates (*n* = 9), and when followed by the same letter do not differ from each other by Tukey's test at *p* < 0.05. (a) Ab-V5; (b) Ab-V6.

The number of *A. brasilense* cells recovered from maize seeds significantly decreased after 24 h of inoculation for both strains, with or without pesticide (Figure 1). In addition, strain Ab-V5, but not Ab-V6, was significantly affected by the seed treatment with the pesticide after 24 h, with a drastic decrease from 4.56 × 10^5^ CFU seed^−1^ in the day of inoculation to 4.37 × 10^2^ CFU seed^−1^ after 24 h. Santos et al. [14] also reported drastic cell mortality of *A. brasilense* after 24 h of exposure to another pesticide (carbendazim + thiram). Pereira et al. [20] observed similar results when exposing these same strains to pesticide-coated (metalaxyl-m + fludioxonil + thiamethoxam + abamectin + Peridiam) maize seeds; after 12 h of exposure, the authors observed a cell survival rate of 65.87% in seeds without pesticide and of 13.56% when treated with the pesticide.

It is important to comment that farmers usually treat their seeds with pesticides and inoculants and sowing may take several hours or even days. Our results indicate that *A. brasilense* is a very sensitive bacterium, with poor survival capacity in maize seed surface, even in the absence of pesticides. Therefore, cell protectors in inoculant formulations, speed of sowing, and other strategies should be searched to guarantee the performance of elite strains of *A. brasilense*.

Neither the inoculation of *A. brasilense* nor the seed treatment with the pesticide affected significantly seed germination rate, ranging from 86.10 to 91.66% (Table 1). Similar results were observed by Vogel and Fey [21]. Dartora et al. [22] also found no effect of the fungicide fludioxonil-metalaxyl and of *A. brasilense* on the percentage of germination of maize seeds. Similarly, in wheat (*Triticum aestivum*), Muraneto et al. [23] observed no differences in germination of seeds treated with fungicide, insecticide, and *A. brasilense*.

For the seed vigor parameters, there was also no interaction between the factors (pesticide and inoculation). However, the best results for total length (36.93 cm), root length (22.66 cm), and shoot length (13.26 cm) were observed in the treatment without pesticide and inoculation (NPWI) treatment. Some farmers have concerns about the inoculation with *Azospirillum* affecting seed germination and seedling growth. Therefore, our data confirm that there was no negative effect caused by strains Ab-V5 and Ab-V6 that could affect plant emergence.

Plant height varied between 67 and 71 cm, culm diameter between 9.6 and 10.7 mm, and shoot dry weight between 2.5 and 3 g, with no significant interaction between the inoculation and the pesticide for any of the parameters (Table 2). Although without statistical difference, the lowest height, culm diameter, and shoot dry mass were observed in the treatment with pesticide and no inoculation (WPNI).

**Figure 2:**
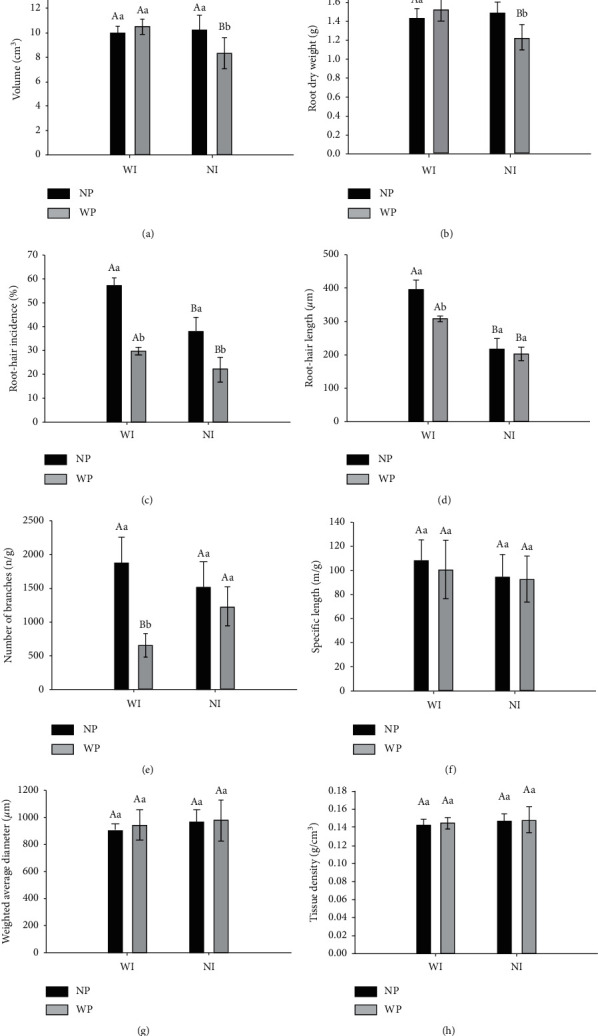
(a) Volume; (b) root dry weight; (c) root hair incidence; (d) root hair length; (e) number of branches; (f) specific length; (g) weighted average diameter; and (h) tissue density of roots of maize whose seeds were treated or not with pesticide and were inoculated or not with *Azospirillum brasilense* strains Ab-V5 + Ab-V6. Plants were grown under greenhouse conditions for 38 days. Data represent the means of five replicates (*n* = 5) and when followed by the same letter do not differ from each other by Tukey's test at *p* < 0.05. Uppercase letters compare means within the same pesticide condition (WP: with pesticide and NP: no pesticide) and lowercase letters compare means within the same inoculation condition (WI: with inoculation and NI: no inoculation). Vertical bars represent the standard deviation.

Even when noninoculated maize seeds were treated with the pesticide, there was a decrease in root volume (Figure 2(a)), root dry weight (Figure 2(b)), and root-hair number (Figure 2(c)); therefore, the stress caused in plants by pesticides can reduce the efficiency of the root system in acquiring water and nutrient resources and, consequently, plant growth. Bonea and Bonciu [24] investigated the cytological and genotoxic effects induced by the fungicide Royal Flo, with thiram in its composition, and verified that the fungicide reduced the mitotic index of the root meristem. In our study, we showed that inoculation with *A. brasilense* helped to mitigate the toxic effects caused by the pesticide on root volume and root dry weight.

The main mechanism by which strains Ab-V5 and Ab-V6 of *A. brasilense* promote plant growth is by the production of auxins that stimulate root development [4, 9]. The auxins are important modulators of cell division, acting on the differentiation and elongation of the root apex, on the initiation and development of secondary roots, and on the development of the vascular system [25]. Indeed, inoculation with *Azospirillum* leads to lateral root promotion [26], helping plant development and increasing the ability to acquire water and mineral nutrients from soil [27]. One of the most pronounced effects of *Azospirillum* inoculation on root morphology is the proliferation of root hairs, thin extensions of root epidermal cells that occur mainly near the apexes of stretching roots [27, 28]. In our study, considering the seeds not treated with the pesticide, the main impact of inoculation with *A. brasilense* was on root-hair incidence (Figure 2(c)) and root-hair length (Figure 2(d)), which are very important properties for the uptake of water and nutrients [27]. However, in the presence of the pesticide, the benefits of inoculation were impaired for these two parameters and also for the number of branches per gram of root (Figure 2(e)).

The increase of root-hair incidence due to inoculation with *Azospirillum* sp. has been reported in other cultures as pearl millet (*Pennisetum americanum* L.) [29], wheat [30, 31], rice (*Oryza sativa* cv.) [32], burr medic (*Medicago polymorpha* L.) [33], alfalfa (*Medicago sativa*) [34], and tomato (*Lycopersicon esculentum* Mill.) [35, 36].

Recently, the impact of inoculation with *A. brasilense* has also been described by Rondina et al. [37] who studied the morphology of soybean roots inoculated with different treatments and observed that inoculation with *Bradyrhizobium* spp. together with *A. brasilense* (Ab-V5 and Ab-V6) increased specific root length, root-hair length, and the number of root branches compared to the single inoculation with *Bradyrhizobium* spp. The authors also reported that the presence of *A. brasilense* inoculated together with *Bradyrhizobium* spp. was determinant for the increase in the percentage of root length with diameter <0.50 mm.

Facing the benefits that can be achieved by the inoculation with *A. brasilense*, particularly with strains Ab-V5 and Ab-V6 [3, 4, 9, 28, 38], nowadays used in more than 7 million doses of inoculants commercialized per year in Brazil, it was mandatory to verify if the treatment of maize seeds with pesticides, broadly adopted by the farmers (e.g., [11]), was compatible with the bacteria. Some of the root parameters evaluated, such as specific length (Figure 2(f)), weighted average diameter (Figure 2(g)), and tissue density (Figure 2(h)), were not affected by the pesticide, neither in inoculated nor in noninoculated plants. However, other parameters such as root-hair incidence (Figure 2(c)), root volume (Figure 2(a)), and root dry weight (Figure 2(b)) were decreased even in noninoculated plants. Noteworthy, for these last two parameters, the inhibition caused by the pesticide was alleviated by the inoculation with *A. brasilense*, highlighting that the bacterium may play a key role in the mitigation of the abiotic stress, as pointed out before for other abiotic stresses, such as salinity [4, 9, 36].

In addition, although we did not find differences in shoot parameters at this early evaluation of plant growth, we should consider that plants were growing under optimal controlled conditions of greenhouse, and that on the field the increase in number of hair and root-hair length in inoculated plants may both decrease the susceptibility to water stress conditions and improve maize nutrition, achieving good productivity results. This is even more important with the increasing number of episodes of drought reported with the global climate change.

## 4. Conclusions

We observed lower survival of *A. brasilense* in the presence of the pesticide Standak™ Top, with an increase in cell mortality after only 24 h of exposure. The treatment of maize seeds with Standak™ Top conferred toxic effects on plants, interfering with their development. Consequently, the known benefits of maize inoculation with *A. brasilense* (e.g., [3, 9, 28]), especially on cell division, differentiation, and elongation of roots, may be impaired by the seed treatment with pesticides. It is important to search for innovative inoculants containing cell protectors (e.g., [39, 40]), or to develop more compatible pesticides, so that the expected benefits of inoculation with plant growth-promoting bacteria can be obtained.

## Figures and Tables

**Table 1:** Germination rate and initial growth of maize seedlings whose seeds were treated or not with pesticide and were inoculated or not with *Azospirillum brasilense* strains Ab-V5 and Ab-V6.

Treatments	Germination rate (%)	Total length (cm)	Root length (cm)	Shoot length (cm)
NPNI	88.88 Aa	33.34 Aa	21.42 Aa	10.91 Aa
WPNI	88.88 Aa	34.15 Aa	20.85 Aa	12.30 Aa
NPWI	86.10 Aa	36.93 Aa	22.66 Aa	13.26 Aa
WPWI	91.66 Aa	35.03 Aa	21.80 Aa	12.23 Aa
*p*-value	0.24	0.90	0.47	0.84
CV (%)	14.06	4.41	4.86	8.97

Data represent the means of five replicates and when followed by the same letter do not differ from each other by Tukey's test at *p* < 0.05. Uppercase letters compare means within the same pesticide condition (WP: with pesticide and NP: no pesticide) and lowercase letters compare means within the same inoculation condition (WI: with inoculation and NI: no inoculation).

**Table 2:** Shoot attributes of maize whose seeds were treated or not with pesticide and were inoculated or not with *Azospirillum brasilense* strains Ab-V5 and Ab-V6.

Treatments	Plant height (cm)	Culm diameter (mm)	Shoot dry mass (g)
NPNI	71.0 Aa	10.4 Aa	3.0 Aa
WPNI	67.0 Aa	9.6 Aa	2.5 Aa
NPWI	67.7 Aa	10.0 Aa	2.7 Aa
WPWI	71.0 Aa	10.7 Aa	2.8 Aa
*p*-value	0.702	0.835	0.0867
CV (%)	5.94%	9.82%	12.84%

Means of five replicates followed by the same letter do not differ from each other by Tukey's test at *p* < 0.05. Uppercase letters compare means within the same pesticide condition (WP: with pesticide and NP: no pesticide) and lowercase letters compare means within the same inoculation condition (WI: with inoculation and NI: no inoculation).

## Data Availability

The data are included in the manuscript. Data with all replicates can be available from the corresponding author upon request.
